# Mental health patient‐reported outcomes among adolescents and young adult cancer survivors: A systematic review

**DOI:** 10.1002/cam4.6444

**Published:** 2023-08-18

**Authors:** Sarah Tanner, Teyl Engstrom, Wen Ray Lee, Cheryl Forbes, Rick Walker, Natalie Bradford, Jason D. Pole

**Affiliations:** ^1^ Centre for Health Services Research The University of Queensland Herston Queensland Australia; ^2^ School of Medicine The University of Queensland Herston Queensland Australia; ^3^ Queensland Children's Hospital Brisbane Queensland Australia; ^4^ Princess Alexandra Hospital Brisbane Queensland Australia; ^5^ Cancer and Palliative Care Outcomes Centre at Centre for Children's Health Research Queensland University of Technology Brisbane Queensland Australia; ^6^ Dalla Lana School of Public Health The University of Toronto Toronto Ontario Canada

**Keywords:** adolescent, cancer, cancer survivors, mental health, patient‐reported outcome measures, routinely collected health data, young adult

## Abstract

**Background:**

Adolescent and young adult (AYA) cancer patients and survivors face significant mental health challenges throughout their cancer journey that are different to those faced by children and older adults. Patient‐reported outcome measures (PROMs) can be used to explore the experiences of AYAs, and to identify important issues and areas for potential improvement in quality of life.

**Objective:**

We aimed to compare patient reported mental health outcomes between AYAs diagnosed with cancer and non‐cancer controls.

**Method:**

We built on a larger systematic review of AYA cancer PROMs which searched PubMed, EMBASE, CINAHL and PsychINFO. This review identified 175 articles, which were filtered to those reporting on mental health and including a non‐cancer control group.

**Results:**

We identified 12 eligible studies. Seven studies (58%) found those diagnosed with cancer reported poorer mental health than the non‐cancer controls. The remaining five (42%) studies found no significant difference in severity or prevalence of mental health between the AYA cancer cohort and the healthy control group. Most (83%) were cross‐sectional studies, highlighting the need for further longitudinal assessment of this group throughout their journey.

**Conclusions:**

The mental health outcomes feature conflicting results and illustrate the need for larger studies to characterise discrepancies.

## 1 INTRODUCTION

Survival rates among adolescent and young adult (AYA) cancer patients are improving in developed countries with 5‐year observed survival rates reaching 85%.^1^, ^2^, ^3^ This means most AYAs diagnosed with cancer will now live for long periods after diagnosis and treatment.^4^ Despite this, cancer remains the most common cause of death from disease among young people.^5^ Because treatment is often intense and toxic, a diagnosis of cancer can still cause significant stress and uncertainty. In Queensland, more than 35,000 people in this age group were diagnosed across three decades, so a significant number of AYAs face these challenges.^5^

Historically, research into cancer patients and survivorship has focused on either children or older adults; AYAs have not been adequately studied or understood.^6^ This population is defined as people aged 15–39.^2^ Further investigation is therefore required to identify the impact a diagnosis has on the lives of AYA cancer patients both around the time of diagnosis and treatment, and during the survivorship period.

AYAs face many challenges as they navigate a phase of their lives when key developmental and life milestones occur. They are often taking on educational challenges; they are developing key relationships and forming their identity. A diagnosis of cancer and the treatment it entails, can disrupt their goals, tear social bonds, affect family lives, lead to increased dependence on caregivers and even lead to the loss of reproductive capacity.^4^, ^7^, ^8^, ^9^

Survivors of AYA cancer are at risk of mental health challenges including anxiety, depression and other mood disorders.^10^ The prevalence of depression in cancer patients as a whole has already been reported to be between 4% and 49% using different assessment methods.^11^, ^12^ More specifically, AYAs are at higher risk than controls of adverse mental health outcomes as measured by outpatient and inpatient interaction with the healthcare system,^13^ and through medical expenditure.^14^ Their scores on mental health questionnaires indicate worse mental health, and they have higher rates of medication use and therapy use.^15^ These measures, however, do not take into account the patient experience, and are instead measures of outputs. We were interested in exploring how this population views its experience, through self‐reporting measures.

One form of investigating outcomes, often used in cancer patients, is through patient‐reported outcome measures (PROMs). PROMs are instruments or tools used to collect information on patient outcomes, as directly reported by the patient through questionnaires.^16^ These allow AYA patients to voice their own experience in an effective and meaningful way, to raise information that may not be otherwise apparent to a clinician. The questionnaires or instruments are often standardised and validated, thereby permitting an assessment over time and between different groups such as peers that have not experienced a cancer diagnosis.

This study builds on the work of a systematic review that identified the PROM domains measured and PROM tools used in AYA cancer patients to understand various outcomes and challenges.^17^ Our research question was, how do mental health outcomes, as measured by PROM tools, differ between AYA cancer patients and survivors and their cancer‐free peers?

## 2 METHODS

An initial systematic review was undertaken, the full details of which are described elsewhere.^17^ In summary, four databases (PubMed, EMBASE, CINAHL, PsycINFO) were searched for manuscripts published between 1st January 2011 and 16th June 2021 that focused on AYAs (ages 15–39 years) who were diagnosed with malignant neoplasms and reported any use of a PROM. A search strategy, developed with a professional librarian, used subject headings and related free text searches.^17^

Inclusion criteria were, AYA focused (either two‐thirds of study cohort aged 15–39 years at diagnosis, or results for this age group reported separately); diagnosed with a malignant neoplasm; and PROM as a central outcome. Publications that reported on PROM validation studies, protocols or outcomes for family or caregivers only were excluded.

Manuscripts first underwent a title and abstract review, conducted independently by two out of three reviewers (TE, ST, WRL). Consensus was obtained via discussion at a team meeting with all three authors. Cohen's Kappa for title and abstract review was 0.59, representing moderate agreement. Manuscripts subsequently underwent a full‐text review, at which time additional exclusion criteria were applied: inability to identify full text; not written in English; or review article. Again, this was undertaken independently by two out of three researchers (TE, ST, WRL). Consensus was again obtained via discussion at a team meeting with these reviewers. Cohen's Kappa for the full‐text review was 0.69, representing substantial agreement. The final papers underwent data extraction for the relevant information.

Manuscripts identified during the data abstraction phase that used a PROM tool to measure a mental health outcome were considered for inclusion in this systematic review. These included—measures of *mental component summary* (MCS), measures of self‐reported *mental health* as an outcome or measures of self‐reported *anxiety* or *depression*. Other components of psychological wellbeing such as distress, stress or emotional function were considered as separate domains, and were therefore not included as part of this review. All identified mental health manuscripts were then re‐reviewed for the specific exclusion and inclusion criteria. Only studies with a cancer‐free AYA comparison group were included. For this review, a cancer‐free AYA comparison group included either a separate control group of cancer‐free peers that were studied, or AYA population norms. Studies using a cross‐sectional comparison between the groups were included regardless of the number of comparison time points. A reverse reference search of all identified manuscripts was undertaken to identify additional relevant studies that may have been missed in the initial review. Finally, the included manuscripts underwent further data extraction which included additional details of the mental health measures along with the outcomes for both AYAs with and without cancer.

Two authors assessed each study for risk of bias using the risk of bias in non‐randomised studies of interventions (ROBINS‐I) tool.^20^ The studies were assessed independently, and any discrepancies were discussed and resolved. Risk of bias was assessed in several domains: confounding, selection of participants, classification of intervention, deviations from intended interventions, missing data, measurement of outcomes and selection of reported results. The possible levels of risk in the assessment were low, moderate, serious or critical. Each domain received a score, and the overall risk of bias of the study was determined by the least favourable score in any domain.

## 3 RESULTS

### 3.1 Study characteristics

**FIGURE 1:**
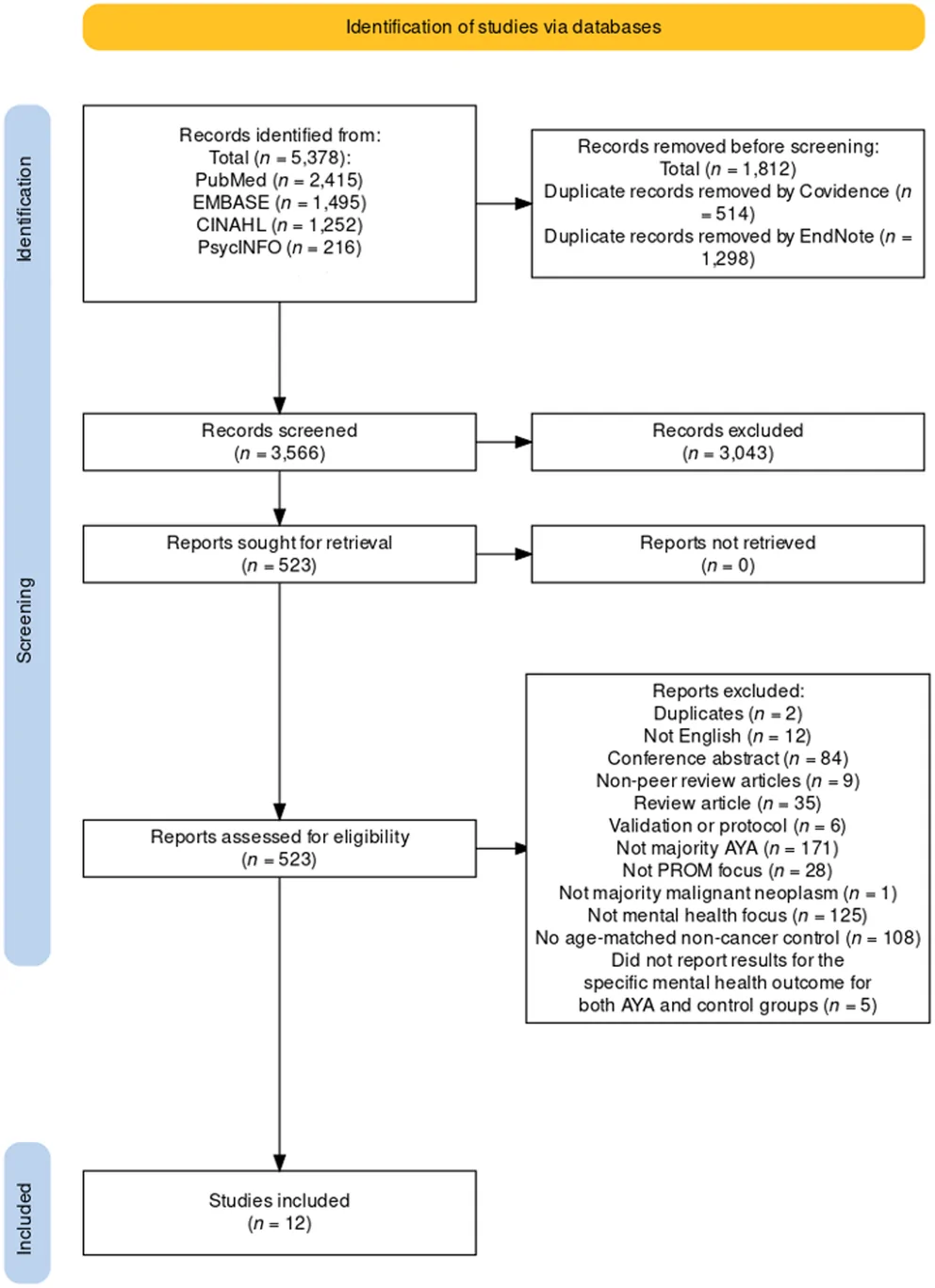
PRISMA study selection diagram.^18^ For more information, visit: http://www.prisma‐statement.org/. Made using online tool by Haddaway et al.^19^

Twelve articles met the inclusion criteria and were included in the final review (Figure 1). A reverse reference search was completed, but no further articles were identified that matched the inclusion criteria. A summary of included study details can be found in Table 1, with full detail in Table 2. One was a cohort study^23^; one was a cross‐sectional cohort study^9^; the other ten were cross‐sectional study designs.^4^, ^6^, ^7^, ^8^, ^21^, ^22^, ^24^, ^25^, ^26^, ^27^ Seven of the 12 were published in 2015 or later^4^, ^7^, ^21^, ^22^, ^23^, ^24^, ^27^; the other five were published between 2011 and 2014.^6^, ^8^, ^9^, ^25^, ^26^ Four of the studies were conducted in a European context.^7^, ^8^, ^21^, ^22^ The other eight were North American studies, with six from the USA^6^, ^9^, ^23^, ^25^, ^26^, ^27^ and two from Canada.^4^, ^24^

**TABLE 1:** Patient‐reported outcome measures (PROMs) measured by included studies.

Author	PROM outcome measured
Bártolo et al.^21^	Anxiety, depression
Harju et al.^22^	Mental component summary
Husson et al.^23^	Mental component summary
Kirchhoff et al.^6^	≥15 vs. <15 days per month of poor mental health
Lang et al.^24^	Self‐perceived mental health
Michel et al.^7^	Anxiety, depression
Monteiro et al.^8^	Anxiety, depression
Salsman et al.^9^	Anxiety, depression
Schulte et al.^4^	Mental component summary, mental health
Smith et al.^25^	Mental component summary
Tai et al.^26^	≥15 vs. <15 days per month of poor mental health
Warner et al.^27^	≥15 vs. <15 days per month of poor mental health

**TABLE 2:** Mental health outcomes in AYA cancer patients and survivors compared with cancer‐free peers.

Study	Study design	Country	Patients	Patient ages mean (SD) or median (IQR)	Sex distribution—patients *N* (%)	Cancer type	Comparison group	Survey	Findings
Bártolo et al. (2020)	Cross‐sectional	Portugal	Breast cancer survivors; *n* = 43	Mean age at study: 36.16 (3.11); at diagnosis: 33.40 (3.81)	Female: 43 (100%); Male: 0; 0%	Breast cancer	Healthy controls and infertile patients; *n* = 37; Mean age at enrolment: 32.41 (3.87) and 35.02 (3.37), respectively	HADS	In cancer and control groups, 25.6% and 27% of women reported moderate to severe anxiety, respectively
									Anxiety and depressive symptoms did not significantly differ between groups
									Most participants scored low on the depression scale
Harju et al. (2018)	Cross‐sectional	Switzerland	AYA cancer survivors; *N* = 155	Mean age at study: 34 (5.87); at diagnosis: 21.6 (2.89)	Female: 59 (38.1%); Male: 96 (61.9%)	CNS, germ cell, renal, hepatic and bone tumours; lymphoma, leukaemia, neuroblastoma; soft tissue sarcoma	Swiss general population, random selection; *n* = 350; mean age: 35.5 (7.21)	SF‐12	Male survivors had better mental health, and female survivors worse, than controls
									Proportion of survivors and controls with poor mental health did not differ
									Women, people with a migration background, the unemployed and individuals with late effects, were more likely to report poor mental health
									Germ cell tumour diagnoses led to better mental health than lymphoma diagnoses
Husson et al. (2017)	Cohort	USA	AYA cancer patients with first diagnosis; *N* = 176 at T1; *N* = 141 at 24‐mo follow‐up	Mean age at study: N/A; at diagnosis: 23.6 (8.9) years	Female: 79 (44.9%); Male: 97 (55.1%)	Any invasive cancer	US population norms—weighted means and pooled standard deviation for the 18–44 years age group	SF‐36	AYA patients reported worse SF‐36 subscale scores except for mental health component, across all three measurement points
Kirchhoff et al. (2014)	Cross‐sectional	USA	Cancer patients and survivors diagnosed in AYA; at least 5 years from diagnosis; *N* = 8375	Mean age at study: N/A; at diagnosis: N/A	Female: 7051 (84.2%); Male: 1324 (15.8%)	Any cancer	US AYAs with no history of cancer; *n* = 334,759	BRFSS	Survivors experienced more poor mental health days per month than control group, irrespective of racial/ethnic group
									Proportions of survivors reporting 15 or more poor mental health days consistently higher than controls
									Younger survivors had worst mental health, peaking at 20‐ to 29‐year‐olds
									Age at diagnosis had an impact, with worse mental health among survivors who were diagnosed at 15–19 years compared with 35–39 years
Lang et al. (2018)	Cross‐sectional	Canada	AYA cancer survivors; *N* = 881	Mean age at study: N/A; at diagnosis: N/A	Female: 609 (69.1%); Male: 272 (30.9%)	Any cancer	Cancer‐free AYAs; *n* = 82,889	CCHS	AYA cancer survivors more often report diagnosis of anxiety and/or mood disorder than healthy peers
									AYA cancer survivors also report poor self‐perceived mental health than cancer‐free peers
Michel et al. (2019)	Cross‐sectional	Switzerland	AYA cancer survivors diagnosed between 16 and 25 years old; *N* = 160	Mean age at study: 34 (5.8); at diagnosis: 21.6 (2.8)	Female: 62 (38.7%); Male: 98 (61.3%)	CNS, germ cell, renal, hepatic and bone tumours; lymphoma, leukaemia, neuroblastoma; soft tissue sarcoma	From Swiss general population 21‐ to 46‐yo; *n* = 358; weighted according to gender distribution of AYA cancer survivors	BSI‐18	21.3% of survivors were considered distressed, compared with 16.6% of controls
									More distressed women (27.4%) than men (17.4%)
									Risk factors for distress included: having a migration background and being unemployed
									Women and people not in in a relationship partnership were more likely to be depressed
									Presence of late effects was a risk factor for distress
Monteiro et al. (2013)	Cross‐sectional	Portugal	University student AYA cancer patients and survivors; *N* = 36	Mean age at study: 28.53 (5.13); at diagnosis: N/A	Female: 24 (66.7%); Male: 12 (33.3%)	Any cancer	University students; *n* = 435; mean age: 28.86 (4.12)	HADS	Levels of anxiety and depression in cancer patients or off‐treatment survivors did not differ from controls. Nor was there a difference between off‐treatment survivors and on‐treatment patients
									Groups separated by age at diagnosis did not differ significantly in terms of anxiety and depression
Salsman et al. (2014)	Cross‐sectional cohort	USA	AYA cancer patients & survivors; *N* = 335	Mean age at study 31.8 (SD = 5.4); at diagnosis: N/A	Female: 229 (68.4%); Male: 106 (31.6%)	Any cancer	US cancer‐free AYAs; *n* = 335; mean age at study: 31.8 (5.4)	MHI‐18	No difference in anxiety, depression or positive affect were found in any of group, cohort, or group‐by‐cohort interactions
									30‐ to 39‐year‐olds reported less anxiety and depression than 25‐ to 29‐year‐olds
									There were significant differences in levels of anxiety and depression on secondary analyses by age group
Schulte et al. (2021)	Cross‐sectional	Canada	AYA cancer survivors >2 years post‐therapy completion; *N* = 195	Mean age at study: 35.62 (6.89); at diagnosis: 27.74 (6.27)	Female: 166 (82.2%); Male: 36 (17.8%)	Any cancer	Age‐ and sex‐matched from Canadian Community Health Survey; *n* = 665	SF‐12	Survivors had worse mental health compared with the comparison group, even when controlled for sex and age
									Worse sleep quality was associated with worse mental health
									Survivors not in a relationship had better mental health than those in a relationship
									None of: sex, current age, income, age at diagnosis, employment status, time since completing treatment, having received surgery or chemotherapy, social support and posttraumatic growth were significantly associated with mental health”
Smith et al. (2013)	Cross‐sectional	USA	AYA cancer patients 6‐ to 14‐months post‐diagnosis; *N* = 523	Mean age at study: N/A; at diagnosis: N/A	Female: 92 (36.7%); Male: 331 (63.3%)	Non‐Hodgkin and Hodgkin lymphoma, germ cell cancer, acute lymphocytic leukaemia or sarcoma	US population norms—age‐matched	SF‐12	Worse outcomes for mental HRQOL in patients who: were unmarried; lacked insurance; had germ cell cancer compared to NHL and ALL; had comorbid conditions; underwent both radiation and chemotherapy (compared to surgery only); and were undergoing current treatment
									Young adults with cancer report lower scores on MCS than population norms
Tai et al. (2012)	Cross‐sectional	USA	Individuals with AYA cancer diagnosed between ages 15–29; *N* = 4054	Median age at study: 40.2 (38.7–41.4)	Female: 3286 (81.1%); Male: 786 (18.9%)	Any cancer	Respondents without history of cancer; *n* = 345,592	BRFSS	A greater proportion of AYA cancer survivors than controls reported 14 or more days of poor mental health in the past month
Warner et al. (2016)	Cross‐sectional	USA	Cancer patients & survivors diagnosed in AYA; at least 5 years from diagnosis; *N* = 7619	Mean age at study: N/A; at diagnosis: N/A	Female: 6441 (84.5%); Male: 1178 (15.5%)	Any cancer	US AYAs with no history of cancer; *n* = 334,759	BRFSS	Female survivors more likely to experience 15 or more days per month of poor mental health than controls, as were male survivors; but more female survivors experienced this than male

Abbreviations: AYA, adolescent and young adult; BRFSS, Behavioral Risk Surveillance System; CCHS, Canadian Community Health Survey; CNS, Central Nervous System; HADS, Hospital Anxiety and Depression Scale.

Three studies used the Short‐Form‐12 Scale (SF‐12).^4^, ^22^, ^25^ Three studies used the nationwide health survey the 2009 Behavioral Risk Surveillance System (BRFSS) survey, instead of a mental health specific tool, to collect information.^6^, ^26^, ^27^ Two studies used the Hospital Anxiety and Depression Scale.^8^, ^21^ One used the Short‐Form‐36 (SF‐36) questionnaire^23^; one used the Brief Symptoms Inventory (BSI‐18)^7^; and one used the Mental Health Inventory (MHI‐18).^9^ A further study similarly relied on the Canadian Community Health Survey (CCHS), which uses the SF‐36.^24^

Four of the studies limited their inclusion criteria to certain types of cancer. One specifically looked at breast cancer.^21^ Two studies included a list of specific cancers Central Nervous System (CNS) tumours, germ cell tumours, lymphomas, leukaemias, neuroblastomas, renal, hepatic and bone tumours, as well as soft tissue sarcomas) to allow for comparison with outcomes for childhood cancer patients.^7^, ^22^ The fourth paper that limited its inclusion criteria to certain types of cancer examined non‐Hodgkin lymphoma, Hodgkin's lymphoma, germ cell cancer, acute lymphoblastic leukaemia or sarcoma.^25^ All other papers (8/12) had broader inclusion criteria, involving a diagnosis of any form of cancer.^4^, ^6^, ^8^, ^9^, ^23^, ^24^, ^26^, ^27^

The complete assessment of the risk of bias among the included studies is presented in Table 3. Overall, the risk of bias assessment deemed seven studies low risk of bias and five studies moderate risk. Five (41.7%) of the papers scored moderate risk for bias due to selection of participants into the study.^4^, ^7^, ^8^, ^23^, ^25^ Two of those studies (16.7% of total) also scored moderate risk for each of bias due to confounding^8^, ^23^ and bias due to deviations from intended interventions.^4^, ^23^

**TABLE 3:** Risk of bias assessment of included studies.

Study		Pre‐intervention		At intervention	Post‐intervention				Overall risk of bias
First Author	Year	Bias due to confounding	Bias due to selection of participants into the study	Bias in classification of interventions	Bias due to deviations from intended interventions	Bias due to missing data	Bias in measurement of outcomes	Bias in selection of the reported result	Severity
Bartolo	2020	Low	Low	Low	Low	Low	Low	Low	Low
Harju	2018	Low	Low	Low	Low	Low	Low	Low	Low
Husson	2017	Moderate	Moderate	Low	Moderate	Low	Low	Low	Moderate
Kirchoff	2014	Low	Low	Low	Low	Low	Low	Low	Low
Lang	2018	Low	Low	Low	Low	Low	Low	Low	Low
Michel	2019	Low	Moderate	Low	Low	Low	Low	Low	Moderate
Monteiro	2013	Moderate	Moderate	Low	Low	Low	Low	Low	Moderate
Salsman	2014	Low	Low	Low	Low	Low	Low	Low	Low
Schulte	2021	Low	Moderate	Low	Moderate	Low	Low	Low	Moderate
Smith	2013	Low	Moderate	Low	Low	Low	Low	Low	Moderate
Tai	2012	Low	Low	Low	Low	Low	Low	Low	Low
Warner	2016	Low	Low	Low	Low	Low	Low	Low	Low

### 3.2 Participant characteristics

The three BRFSS papers studied 8375,^6^ 7619,^27^ and 4054^26^ participants, respectively. All three included all types of cancer, except for non‐melanomatous skin cancers. Because each study selected participants from the same main data source, the nation‐wide 2009 BRFSS study, there is likely a significant overlap among subjects across the papers. Assessing the exact overlap is difficult given each study had slightly different exclusion and inclusion criteria; the third study, for example, included US territories while the other two did not, and it also restricted its analysis to the 15–29 age group. The inclusion and exclusion criteria for the first two studies were very similar but not identical. These differences explain the discrepancy in the number of participants that were included in each study.

Due to the overlap in samples, the following patient characteristics will only be reported from the nine non‐BRFSS studies. In total, 2974 participants were included in the other studies. This includes participants who were AYA cancer patients at the time of the study, and individuals who were longer term survivors following a diagnosis of cancer in their AYA years.

Four studies reported mean age both at diagnosis and time of study.^4^, ^7^, ^21^, ^22^ Two studies reported only mean age at study^8^, ^9^; one reported only mean age at time of diagnosis^23^; one study reported median age at time of study.^26^ The two studies that reported only mean age at time of study focused on patients at or close to the time of treatment, instead of survivors, so the ages at study and diagnosis would be close together. Of the studies reporting the mean age at diagnosis, in fourth the mean age was in the 20s, ranging between 21.6 and 27.7 years.^4^, ^7^, ^22^, ^23^ In the fifth, the mean age was 33.4 years.^21^ In the six studies reporting mean age at time of study, the mean age was between 28.5 and 36.2 years old.^4^, ^7^, ^8^, ^9^, ^21^, ^22^

The sex of participants was reported in all studies. There were a total of 1821 (61.2%) women and 1153 (38.8%) men.

### 3.3 Control characteristics

Two of the studies used country specific population norms—one was age‐matched,^25^ the second used weighted means and pooled standard deviations for the 18–44 age group.^23^ Four papers used large population‐based studies that included both cancer and non‐cancer respondents. Three used the BRFSS as described above, and one used the CCHS. The three BRFSS papers reported on a total of 334,759,^6^ 345,592^26^ and 334,759^27^ controls, respectively, while the CCHS included 82,889 controls.^24^ The remaining six papers had a total of 2180 controls.^4^, ^7^, ^8^, ^9^, ^21^, ^22^ In four of these six papers, mean age of controls was reported,^8^, ^9^, ^21^, ^22^ and ranged between 28.9 and 35.5 and in five of them, sex was reported,^4^, ^8^, ^9^, ^21^, ^22^ with a total of 1285 women and 536 women.

### 3.4 Mental health outcomes

#### 3.4.1 Outcomes

Five studies found no significant difference in severity or prevalence of anxiety and depression between the AYA cohort and the healthy control group.^8^, ^9^, ^21^, ^22^, ^23^ In one of these studies the AYA cohort included patients and survivors, covering from 0 to 60 months post‐treatment.^9^ A second of these studies focused on patients,^23^ with the remaining three studies looking at survivors only.^8^, ^21^, ^22^

The three studies that used the BRFSS survey data all found that the number of poor mental health days per month was higher in survivors of AYA cancer than in control groups.^6^, ^26^, ^27^ Kirchhoff et al.found a statistically significant incidence rate ratio of poor mental health days per month for survivors compared with the control group of 1.66 (95% CI: 1.55–1.79, *p* < 0.001).^6^ In this study, 17.2% of survivors compared to 10.0% of controls experienced poor mental health for more than half the month.^6^ Similarly, Tai et al. found those numbers to be 19.5% in survivors and 10.2% in controls with no cancer history.^26^

Three studies identified that survivors reported worse mental health, in terms of both self‐perception of mental health and in anxiety and mood disorders diagnosed by a healthcare professional.^4^, ^7^, ^24^

A further study found that the AYA patients scored statistically significantly lower (indicating worse mental health) than the AYA‐specific age‐matched US norm on the mental health aspect of the SF‐12 survey, but only in the age groups of 25–34 and 35–41 year‐olds, and not among 18–24 year‐olds, where a statistically significant difference was not found.^25^ Similar results were found in the MCS, which was statistically significantly worse in 25‐ to 34‐ and 35‐ to 41‐year‐old cancer patients, but not in the 18‐ to 24‐year‐old age group.^25^

#### 3.4.2 Associations between mental health and other factors

Three studies compared mental health outcomes between males and females, focused on survivors of AYA cancer.^7^, ^22^, ^27^ One study found that mental health in male survivors was better than in controls, while among female survivors, mental health was worse (OR = 3.74, *p* = 0.001).^22^ Similarly, another study reported female survivors were more likely than male survivors to be depressed based on the BSI‐18, with an odds ratio of 2.9 (*p* = 0.008).^7^ A third identified that more female survivors reported greater than 15 days of poor mental health than male survivors.^27^

Sociodemographic factors that were associated with worse mental health in female survivors included having a migration background,^22^ or being unemployed.^22^ These factors were found to be statistically significant in both univariate and multivariate regression.^22^ Another study found these associations were significant in both male and female survivors.^7^ Interestingly, not being married or in a relationship was found to be a risk factor for depression in the patient^25^ and the survivor cohort,^7^ but this was contradicted by another study that found mental health was better for those survivors not in a relationship.^4^

The impact of age on mental health outcomes in this group compared to cancer‐free peers was also examined and the findings suggest that the 20‐ to 29‐year‐old age group is the most vulnerable. One study found that age played a role, with the poorest mental health found in survivors aged 20–29 years old.^6^ 24% of the survivors in that age group, compared with 10.8% of the control group, reported at least 15 days per month of poor mental health.^6^ This was supported by a second paper suggesting that in the 30‐ to 39‐year‐old age group, there was significantly less anxiety and depression compared with the 25‐ to 29‐year‐old age group.^9^ Age at diagnosis was similarly relevant: survivors diagnosed at age 15–19 were more likely than those diagnosed at ages 35–39 to have ≥15 days of poor mental health, with prevalence of 25.0% and 14.6%, respectively.^6^ This was not a unanimous finding, however, as other studies found that age at diagnosis was not significantly different in terms of anxiety and depression.^8^

Health and cancer factors were also found to affect mental health in AYAs. Tumour type, for example, made a difference, with one study finding that patients with germ cell tumours were more likely to have favourable mental health outcomes than lymphoma patients.^22^ This was not a unanimous finding, however, with Smith et al. finding the opposite.^25^ Additionally, patients suffering from late effects (such as infertility, endocrine complications or other somatic or psychological complaints) were unsurprisingly more likely to have poor mental health outcomes, with odds ratios of 3.0 (*p* = 0.021) for depression and 3.1 (*p* = 0.010) for anxiety,^7^ and 8.02 (*p* < 0.001) overall.^22^ The impact of late effects on anxiety or depression was also found to be statistically significant in multivariable and univariable regression.^7^ One study reported that survivors with poor sleep quality were more likely to report poor mental health.^4^ AYA patients receiving chemotherapy and radiation therapy had worse mental health than those who only underwent surgery, as did those who were still receiving treatment.^25^ These findings were not unanimous, however, with other studies finding that there was no difference between therapy type^4^ or whether treatment was current.^8^ Interestingly, one study found that none of the characteristics of sex, current age, age at diagnosis or employment status was significantly associated with poorer mental health in survivors,^4^ also contradicting the previously described findings.

## 4 DISCUSSION

In this systematic review of 12 studies, we provided a comprehensive overview of self‐perceived mental health among AYA cancer patients and survivors compared with healthy peers. A small majority of these studies (7/12) found statistically significantly worse mental health outcomes among the cancer cohort compared to their cancer‐free peers; the remaining five studies did not find a statistically significant difference in mental health between the two groups. The paucity of studies in this area and the inconsistent findings leave a sense that more research is needed to confirm the nature of AYA cancer survivor and patient‐reported mental health. Furthermore, most of the research identified by this systematic review was cross‐sectional; there is scope for further work in the form of longitudinal and longer term studies.

Despite the broad inclusion criteria of the initial study, only a single longitudinal cohort study was identified that measured mental health over time in this group of interest. It followed patients only in the first 2 years of diagnosis, and not over the longer term.^23^ Interestingly, this study found that mental health improved over time, but at no point was it significantly different from the normative population.^23^ The improvement in mental health during a period following diagnosis has been reported elsewhere, including Kwak et al., who noted it to occur over the course of a year.^28^ In that study, anxiety and depression were at their peak at the time of diagnosis.^28^ A nadir occurred at the 6‐month mark, where the presence of both symptoms fell, followed by a slight increase at the 12‐month point. However, it remained lower at this point than at time of diagnosis.^28^

A recent systematic review into adolescent and AYA cancer survivors over the long term identified five longitudinal studies that investigated psychological health as a primary outcome, and two that investigated it as a secondary outcome.^29^ Many of their identified papers did not compare AYAs with healthy controls; and with two of the primary outcome papers published before 2010, there is a lack of more contemporary research. Ultimately, there is a distinct lack of longer term follow‐up and comparison to show the evolution of mental health in this population over time—especially longer periods—compared with healthy peers. More longitudinal research is needed to characterise the time course.

We identified three papers that reported statistically significantly worse mental health and increased rates of anxiety and mood disorders in AYA survivors in a study population, compared with cancer‐free controls. Covering more than 1200 cancer survivors, these papers had a substantial sample size. This is supported by a further three papers that used the BRFSS data to illustrate that survivors are more likely to have ≥15 days of poor mental health per month than cancer‐free peers. The remaining four studies that covered AYA survivors found no statistically significant difference in mental health between survivors and controls. No studies found a difference in the other direction to suggest that AYA survivors may have better mental health than their cancer‐free counterparts. Overall, then, the evidence favours the idea that AYA survivors have poorer mental health outcomes than their cancer‐free peers, though this area would benefit from further comparative research.

It was interesting that AYA patients receiving chemotherapy and radiation therapy had worse mental health than those who only underwent surgery in one study,^25^ though a second study found no difference.^4^ This is possible that this is a result of the systemic effects of chemotherapy and radiation therapy, as well as the more protracted treatment course required with both therapies compared with a potentially one‐off operation.^30^ An association between chemotherapy and worse quality of life has been found in another study into breast cancer survivors.^31^

The systemic effects of chemotherapy and radiation therapy could also explain the association between being still on treatment and having worse mental health outcomes compared with survivors found by that paper,^25^ though again it was not a finding across the board with another study not finding any difference between the two.^8^ Perhaps survivorship leads to a sense of having “beaten” cancer, and having overcome a significant adversity, whereas still undergoing therapy means the adversity remains present.

Small sample sizes and the selection of study participants indicates that more rigorous research with larger studies would be of benefit. The three studies that found no difference in levels of anxiety or depression between the AYA group and the control group had small sample sizes, with 43, 36 and 335 participants. Furthermore, in one of these studies, participants were selected from a university survey. Cancer survivors who can attend university may be higher functioning in everyday life, and have a lower residual burden, such that they can cope with the demands of university. As a result, it is possible they are not representative of the wider population of AYA cancer patients and survivors.

The incongruence discovered with these outcomes illustrates the importance of this review. Such discrepancies demonstrate the need to further characterise the mental health outcomes of, and areas of difficulty faced by, AYAs with cancer compared to their healthy peers.

All three studies that compared both sexes showed women having poorer mental health outcomes than men and being more likely to experience anxiety and depression. This finding is in line with research that has identified that female cancer patients report the lower quality of life outcomes compared to their male counterparts.^30^ In that study, the difference in quality of life outcomes was found across the board in multiple measures of health‐related quality of life (HRQOL) and PROM domains. Interestingly, the difference between male and female survivors is more pronounced than in the age‐matched general population.^30^ Geue et al. posited that the higher levels of insomnia in female cancer patients may be related to higher levels of emotional distress, and that there are gender differences in emotional expression that may be the cause.^30^ The same could be said for mental health in general.^30^ The same authors hypothesised that the lower HRQOL may also be due to different medical treatments as a result of different cancer types and distributions, which is also possible in the case of mental health.^30^

Several studies found that age played a role in poorer mental health outcomes, though the findings were not unanimous. Our results overall suggest that there is a significant period of mental health vulnerability in the early AYA period that is not as prominent in the later years. More research would be useful to further characterise the differences in age groups.

### 4.1 Limitations

This review has some limitations. First, the scope of mental health for the purposes of the review was specific; for example, it did not encompass wider definitions of stress, or psychological concepts such as growth or life outlook. Those aspects were coded differently in the scoping review and may be subject to a systematic review of their own.

Furthermore, we limited our search to full‐text English language articles. Studies in other languages may contribute additional knowledge but was missed by this search exclusion.

The use of population norm values as a control group by a handful of the studies is a further limitation. Research has found that it is difficult to compare the SF‐36 responses of a group with an illness to a population normative data.^32^ This is because the group of interest can have skewed or unusual distributions.^32^ It is also difficult to make gender and age comparisons.^32^ However, it was necessary for us to do so to include more studies in our review and therefore obtain more valuable data on mental health outcomes in AYAs.

### 4.2 Summary

Overall, there is little consensus as to whether AYA cancer patients have worse mental health outcomes than their healthy peers. It may be that the mental health challenges in all people in this age group are so significant that the addition of a cancer diagnosis, followed by improvement and recovery from cancer, does not add a greater burden. There is a gender difference, with most studies finding that women are worse off than men. There is an age element as well, with younger patients more at risk. Young women are a particularly vulnerable group and may benefit from further investigation into how these poor mental health outcomes can be improved. There is also scope for more longitudinal research into this vulnerable group, to further characterise their mental health challenges and experiences and improve outcomes in this population.

## AUTHOR CONTRIBUTIONS

**Sarah Tanner:** Data curation (equal); formal analysis (equal); methodology (equal); writing – original draft (equal); writing – review and editing (equal). **Teyl Engstrom:** Data curation (equal); investigation (equal); methodology (equal); project administration (equal); writing – review and editing (equal). **Wen Ray Lee:** Data curation (equal); formal analysis (equal); writing – review and editing (equal). **Cheryl Forbes:** Data curation (equal); formal analysis (equal); writing – review and editing (equal). **Rick Walker:** Investigation (equal); writing – review and editing (equal). **Natalie Bradford:** Investigation (equal); writing – review and editing (equal). **Jason D. Pole:** Conceptualization (equal); investigation (equal); methodology (equal); supervision (equal); writing – review and editing (equal).

## FUNDING INFORMATION

The authors received no specific funding for this work.

## CONFLICT OF INTEREST STATEMENT

The authors declare no potential conflicts of interest.

## PRECIS FOR USE IN THE TABLE OF CONTENTS

We identified twelve eligible studies, seven (58%) of which found those diagnosed with cancer reported poorer mental health than the non‐cancer controls; the remaining five (42%) studies found no significant difference in severity or prevalence of mental health between the groups. The mental health outcomes feature conflicting results and illustrate the need for larger studies to characterise discrepancies.

## Data Availability

Data sharing not applicable—no new data generated, or the article describes entirely theoretical research.
